# Pulmonary embolism following endovascular aortic repair for a ruptured abdominal aortic aneurysm: A case report

**DOI:** 10.1016/j.ijscr.2025.111685

**Published:** 2025-07-15

**Authors:** Shunsuke Taguchi, Shun Nakaji, Ichiro Matsumaru, Kazuki Hisatomi, Hiromitsu Teratani, Takashi Miura

**Affiliations:** a Department of Cardiovascular Surgery, Nagasaki University Hospital, Japan

**Keywords:** Rupture, Abdominal aortic aneurysm, Pulmonary embolism, Deep venous thrombosis

## Abstract

**Introduction and importance:**

The primary complications of ruptured abdominal aortic aneurysms (AAAs) include abdominal compartment syndrome, ischemic enteritis, cardiac complications, and lower limb ischemia. Pulmonary embolisms (PEs) resulting from deep venous thrombosis (DVT) are uncommon. Herein, we report an important case in which a critical PE developed in a patient after emergency endovascular aortic repair (EVAR) for a ruptured AAA. Notably, the patient's life was saved through cardiac surgery without compromising his activities of daily living.

**Presentation of case:**

A 64-year-old man was admitted to our hospital with a ruptured AAA and a massive right retroperitoneal hematoma. We performed emergency EVAR, and an open abdomen and temporary abdominal closure technique. On post-operative day 5, he experienced sudden cardiac arrest due to PE caused by DVT. An emergency pulmonary artery thrombectomy was performed. The abdomen was opened again and the retroperitoneal hematoma was removed. The postoperative course was uneventful and the patient was discharged without sequelae.

**Clinical discussion:**

The thrombus formation was considered to have been caused by an aneurysm and a retroperitoneal hematoma that compressed the inferior vena cava.

**Conclusion:**

If a ruptured AAA protrudes toward the right side and is associated with a large right-sided hematoma, clinicians might have to consider removing the hematoma simultaneously via EVAR.

## Highlights

• Ruptured abdominal aortic aneurysms with extensive retroperitoneal hematoma, particularly when the aneurysm extends to the right side, may compress the inferior vena cava and iliac vein.
• Large venous thrombosis caused by compression may lead to a life-threatening pulmonary embolism.
• Retroperitoneal incision and hematoma removal should be considered when selecting endovascular aortic repair for ruptured abdominal aortic aneurysms.
• Careful monitoring of fibrinolytic markers is necessary and early examination is required for patients with high values, even in those who are asymptomatic.

## 1 Introduction

The main complications of ruptured abdominal aortic aneurysms (AAA) are abdominal compartment syndrome, ischemic enteritis, cardiac complications, and lower limb ischemia. While there have been a few reports of deep venous thrombosis (DVT) as a postoperative complication of ruptured AAA, critical pulmonary embolism (PE) following rupture is a rare occurrence. In this report, we describe a case in which critical PE developed in a patient after emergency endovascular aortic repair (EVAR) for a ruptured AAA; however, the patient's life was saved through cardiac surgery without compromising his activities of daily living. This case report has been reported in the line with the SCARE 2025 criteria [1].

## 2 Presentation of case

**Fig. 1:**
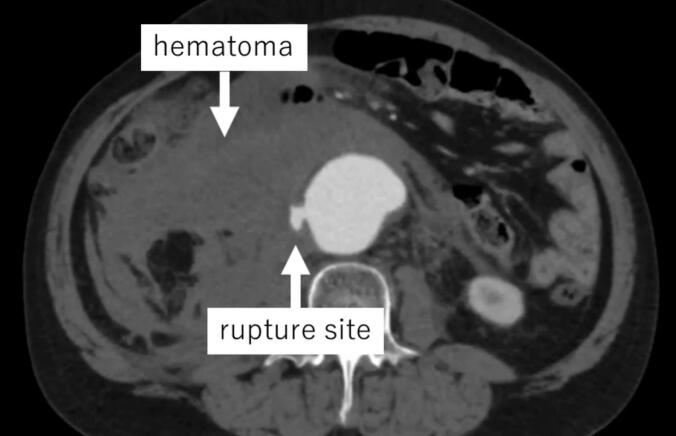
Contrast-enhanced computed tomography revealed a ruptured abdominal aortic aneurysm with a massive right retroperitoneal hematoma.

A 64-year-old man was admitted to our emergency department with sudden abdominal pain and hypotension (systolic blood pressure 70 mmHg). As a university hospital, most patients are referred here from other medical institutions. The patient had no medical history, was not taking any medications, and had no allergies or family history. Contrast-enhanced computed tomography (CT) revealed a ruptured AAA with a diameter of 54 mm and a massive right retroperitoneal hematoma (Fig. 1). There were no significant signs of DVT in the patient's legs or on CT images. The patient's fibrin degradation product (FDP) and D-dimer levels were 5.5 μg/dL and 2.2 μg/dL, respectively.

We performed emergency EVAR using an Excluder stent graft (W. L. Gore & Associates, Inc., Flagstaff, AZ, USA) because priority was given to immediate primary repair. In addition, an open abdomen and temporary abdominal closure technique was performed with the ABTHERA open abdomen negative pressure therapy system (Kinetic Concepts, Inc., San Antonio, TX, USA). This is because development of abdominal compartment syndrome was expected due to the massive hematoma and transfusion load causing bowel wall edema and paralytic ileus.

The postoperative recovery was favorable. The abdomen was closed 3 days after EVAR, and mechanical ventilation was discontinued on postoperative (POD) day 4. Since there was no bleeding into the ABTHERA system or progression of anemia, intravenous heparin was administered at 5000 units/day from POD 2 to prevent DVT due to prolonged bed rest, and the dose was increased to 10,000 units/day on POD 4. During this period, the patient's FDP and D-dimer levels increased significantly (POD 4: 141.4 μg/dL and 71.8 μg/dL; POD 5: 334.6 μg/dL and153.3 μg/dL, respectively). The patient was closely monitored; there was no apparent lower limb swelling, redness, or other findings suggestive of DVT. No imaging studies were performed because the elevated fibrinolytic markers were considered to be a response to the hematoma. On POD 5, he suddenly lost consciousness during repositioning, with hypoxemia, hypotension, and cardiac arrest.

**Fig. 2:**
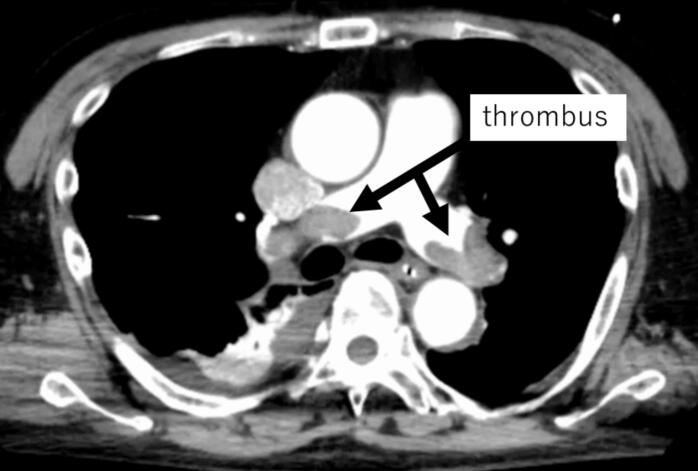
Contrast-enhanced computed tomography revealed embolisms in the main trunks of both pulmonary arteries.

Transthoracic echocardiography revealed right ventricular enlargement, left ventricular collapse, and thrombus-like structures in the right atrium that were suggestive of PE. Spontaneous circulation was achieved by chest compressions and adrenaline administration. Venoarterial extracorporeal membrane oxygenation (V-A ECMO) was immediately established due to the unstable circulation. Contrast-enhanced CT was performed to evaluate for a possible PE and AAA re-rupture. The scan revealed embolism in the main trunks of the bilateral pulmonary arteries and a thrombus in the inferior vena cava (IVC) (Fig. 2).

Given the patient's hemodynamic instability and massive PE, thrombectomy was indicated. An emergency pulmonary artery thrombectomy was performed (Video 1). Because thrombus formation was considered to have been caused by the aneurysm and retroperitoneal hematoma that compressed the IVC, the abdomen was opened again, the retroperitoneal hematoma was removed, and the patient returned to ABTHERA treatment.

The patient's postoperative course was uneventful. The abdomen was successfully closed, and V-A ECMO was discontinued 3 days after reoperation. The FDP and D-dimer levels showed a rapid decreasing trend (post re-operative day 2: 37 μg/dL and 15.5 μg/dL; day 6: 27.3 μg/dL and 11.9 μg/dL, respectively). The patient was discharged without sequelae 42 days after the EVAR. Although a residual DVT was observed in the right lower limb, the thrombus remained stable. The patient continued receiving oral rivaroxaban 15 mg/day. Thereafter, the DVT showed a tendency to shrink without recurrence of PE. Anticoagulation therapy was feasible and appropriate; therefore, an IVC filter was considered unnecessary.

## 3 Discussion

Virchow's triad describes the three fundamental risk factors of thrombosis, comprising vessel wall damage, stasis, and hypercoagulability. There have been a few reports of venous thrombosis due to the compression of the IVC by an unruptured AAA [2,3], in which direct venous compression of the aneurysm contributed to thrombus formation. These reports suggest that an AAA can cause external compression of the IVC, leading to venous stasis. In the present case, in addition to the AAA, a hematoma extended to the right side, which further increased the pressure on the IVC.

DeMaistre et al. reported that DVT may occur more frequently after open surgical repair (10.2 %) than after EVAR (5.3 %) for AAA treatment [4]. Furthermore, Niina et al. reported 1021 cases of symptomatic venous thromboembolism (VTE) in patients with AAA. The high-risk period for VTE lasted approximately 3 months, and during that time, VTE occurrence was highest in patients with coronary disease (2.5 %), after open repair (2.4 %), and in an urgent or emergency setting (2.6 %), whereas the rate was low after endovascular aneurysm repair (1.0 %) [5]. For many patients, EVAR appears favorable with regard to the occurrence of VTE. However, there are few reported cases of EVAR performed in patients with ruptured AAA followed by PE due to venous thrombosis, which is considered uncommon [6]. In a retrospective study of patients undergoing EVAR for ruptured AAA, 18 % (4/22) had massive hematomas (Fitzgerald classification 2–4) that caused DVT, while none of the patients presented small localized hematomas (0 %, 0/9) [7].

These prior reports highlight the importance of addressing the complications associated with residual hematomas after EVAR. Abdominal compartment syndrome has been recognized as a postoperative limitation of EVAR in patients with ruptured AAA; therefore, our patient was managed with an open abdomen. Notably, the risk of thrombosis has not been considered, and in one reported patient with PE following EVAR for a ruptured AAA, a hematoma occurred on the right side of the AAA that compressed the IVC^6^. Similarly, in the present patient, if a ruptured AAA protrudes toward the right side and is associated with a large right-sided hematoma, clinicians might have to consider removing the hematoma simultaneously via EVAR.

The present patient also exhibited markedly elevated FDP and D-dimer levels. These markers are utilized as both negative and positive predictive biomarkers for VTE [8], as well as diagnostic biomarkers for disseminated intravascular coagulation [9]. Furthermore, elevated FDP and D-dimer levels have been reported to indicate massive bleeding in patients undergoing major surgical procedures [10]. In the present case, imaging evaluation was not performed because the elevated FDP and D-dimer levels were presumed to be attributable to the hematoma, given the absence of obvious swelling, redness, and other clinical findings in the lower extremities. As previously noted, the anatomical risk for VTE was elevated, and either venous ultrasonography of the lower extremities or contrast-enhanced CT should have been considered.

## 4 Conclusion

In patients with ruptured AAA accompanied by extensive retroperitoneal hematoma—particularly when the aneurysm protrudes to the right and may compress the IVC or iliac vein—retroperitoneal incision and hematoma evacuation may be considered in selected cases of EVAR. Fibrinolytic marker monitoring should be considered, and early imaging evaluation may be warranted, in asymptomatic patients with markedly elevated levels, although further studies are needed to establish clear guidelines.

The following is the supplementary data related to this article.

Video 1 Video 1

## Author contribution

Shunsuke Taguchi: perform surgical procedure, manuscript editing and writing.

Shun Nakaji: perform surgical procedure, manuscript editing.

Ichiro Matsumaru: manuscript reviewing.

Kazuki Hisatomi: manuscript reviewing.

Hiromitsu Teratani: manuscript editing.

Takashi Miura: perform surgical procedure, supervision.

## Consent

Written informed consent was obtained from the patient for publication of this case report and accompanying images. A copy of the written consent is available for review by the Editor-in-Chief of this journal on request.

## Ethical approval

This case report is based on medical care that was provided as part of routine clinical practice, with no additional interventions conducted for research purposes. The information has been anonymized to ensure that the patient cannot be identified, and privacy has been strictly protected. Therefore, this report does not fall under the scope of activities requiring formal ethical review, and ethical approval is exempted in our Nagasaki University Hospital.

## Guarantor

Shunsuke Taguchi.

## Research registration number

Not applicable.

## Funding

There are no sources of funding for this case report.

## Conflict of interest statement

The authors declare that no conflicts of interest exist related to this study or its publication.
